# Investigation of the Role of a Zinc Uptake Regulator (Zur) in the Virulence of *Pectobacterium odoriferum*

**DOI:** 10.3390/ijms24129991

**Published:** 2023-06-10

**Authors:** Changlong Chen, Shuang Cui, Jiantao Guan, Yanyan Su, Xucong Liang, Yu Tian, Hua Xie

**Affiliations:** 1 Institute of Biotechnology, Beijing Academy of Agriculture and Forestry Sciences, Beijing 100097, China; chenchanglong@baafs.net.cn (C.C.); cuishuang1227@163.com (S.C.); guanjiantao@caas.cn (J.G.); suyanyan5566@163.com (Y.S.); lxcong319@163.com (X.L.); tianyu@baafs.net.cn (Y.T.); 2 Institute of Vegetables and Flowers, Chinese Academy of Agricultural Sciences, Beijing 100081, China

**Keywords:** *Pectobacterium* spp., zinc-uptake regulator, virulence, flagellum, cell motility, metal-ion transport

## Abstract

*Pectobacterium* spp. infect many horticultural crops worldwide and lead to serious crop losses. Zinc-uptake-regulator (Zur) proteins are present widely in prokaryotes and play an important role in pathogenicity. To uncover the role of Zur in *P*. *odoriferum*, we constructed mutant (*ΔZur*) and overexpression [Po (*Zur*)] strains of a *Zur*, and a virulence assay showed that the Po (*Zur*) was of significantly lower virulence, while the *ΔZur* displayed significantly increased virulence on Chinese cabbage compared to their respective control strains, wild-type *P*. *odoriferum* (Po WT) and *P*. *odoriferum* harboring an empty vector (Po (EV)) (*p* < 0.05). The growth curves of the *ΔZur* and Po (*Zur*) showed no obvious differences from those of the control strains. Comparative transcriptome analysis showed that *Zur* overexpression in *P*. *odoriferum* induced differentially expressed genes (DEGs) related to flagellum and cell motility, while mutating *Zur* resulted in DEGs mainly corresponding to divalent-metal-ion transport and membrane transport. Phenotypic experiments on the Po (*Zur*) showed that flagellum numbers and cell motility were reduced in comparison with the control, while those of the *ΔZur* did not change. Collectively, these results show that the Zur negatively regulates the virulence of *P*. *odoriferum* and might function via a dual mechanism dependent on dose.

## 1. Introduction

Zinc (Zn) is the second most abundant transition-metal element among all the essential trace elements in living organisms after iron [1]. It is a cofactor of many enzymes and helps to maintain the structural stability of even more proteins, including transcription factors. Zinc has both structural and functional roles in prokaryotes by binding to 5–6% of proteins [2]. Deficient zinc levels prevent normal cellular function, but excess intracellular zinc can lead to toxicity [3]. This is mainly because it binds to metalloproteins normally associated with other metals from the Irving–Williams series and impairs their function. This phenomenon is called mismetallation [4]. Therefore, intracellular-zinc concentrations must always be carefully regulated through zinc-homeostasis systems to control zinc uptake and release and maintain adequate cellular-zinc concentrations [5].

The zinc-uptake-regulator (Zur) protein belongs to the ferric-uptake-regulator (Fur) family of metalloregulatory proteins. The Zur is present widely in prokaryotes, and it plays an important role in regulating zinc homeostasis [6]. Two main zinc transporters for zinc uptake exist in most gram-negative bacteria: the *znuABC*, a high-affinity zinc-transport system and the low-affinity ZIP family Zn transporter ZupT [7]. *znuABC* is composed of the soluble periplasmic binding protein ZnuA, the high-permeability transmembrane protein ZnuB, and the ATPase ZnuC, and it is regulated by the Zur [8]. Corresponding to the critical role of the Zur in regulating zinc homeostasis, since the first report of the Zur in *Escherichia coli* [9], members of the Zur family have been studied in a wide range of bacteria, including *Bacillus subtilis* [10,11,12], *Streptococcus* sp. [13], *Listeria monocytogenes* [14], *Staphylococcus aureus* [15], *Salmonella enterica* [16], *Yersinia pestis* [17], *Streptomyces coelicolor* [18], *Enterococcus faecalis* [19], *Xanthomonas campestris* [20], *Mycobacterium tuberculosis* [21], and *Pseudomonas aeruginosa* [22].

In addition to its role in regulating zinc homeostasis extra- and intra-cellularly, the Zur has been reported to regulate virulence in several pathogenic bacteria, such as *S*. *enterica* [16], *X*. *campestris* [20], *X*. *oryzae* pv. *oryzae* [23], and *L*. *monocytogenes* [24,25,26]. In plant pathogenic bacteria *X*. *campestris* pv. *campestris*, a Zur protein (XcZur) was demonstrated to be essential for zinc homeostasis and full virulence [20]. When the *XcZur* was mutated, the mutant failed to grow in a rich medium and a non-rich medium supplemented with Zn^2+^ at concentrations of 400 µM and 110 µM, whereas the wild-type strain grew well in the same conditions. In a rich medium with 400 µM Zn^2+^, significantly more Zn^2+^ was accumulated in the mutant than in the wild-type strain. The *XcZur* mutant also showed a reduced virulence on the host plant, Chinese radish (*Raphanus sativus* L. var. radiculus Pers.) and produced less extracellular polysaccharide (EPS) than the wild-type strain. The XcZur activates the expression of the zinc-export gene, XC2976, and represses the expression of the zinc-uptake genes, XC0267, XC2472, and XC3788, to regulate the intracellular zinc level. Particularly, XC3788 shares 38% similarity with the ZnuC, a component of a high-affinity zinc-uptake system *znuABC* [24]. In addition, the XcZur is involved in hypersensitive response (HR) and positively regulates the transcription of genes associated with HR and pathogenicity [25]. In *X*. *oryzae* pv. *oryzae*, the loss of function of the *Zur* revealed that it controls zinc and iron homeostasis and oxidative stress, and the mutation of the *Zur* led to attenuated disease symptoms on the host rice plant and produced reduced amounts of EPSs [23].

*Pectobacterium* spp. are distributed worldwide and cause soft rot disease on various economically important vegetables, fruit, and ornamental plants. They result in serious crop losses during the cultivation, harvest, storage, transit, and marketing processes and are among the top 10 plant pathogenic bacteria on grounds of economic damage [27,28,29]. *Pectobacterium odoriferum* is one of the 20 species in the *Pectobacterium* genus [30,31]. Typically, the coordinated production of a large arsenal of plant-cell-wall-degrading enzymes (PCWDEs) by *Pectobacterium* spp. represents an important determinant of pathogenicity [32,33]. In addition, minor virulence proteins, such as Nip and Svx, as well as the structural proteins of flagella and type III (T3SS) and type VI (T6SS) secretion systems, are also involved [34]. However, mechanisms underlying the pathogenicity of *P. odoriferum* are largely unknown, including the role of the Zur in *P*. *odoriferum*.

In the present study, the *Zur* gene was first identified in *P*. *odoriferum*. Subsequently, we generated *Zur*-knockout mutant and overexpression strains, and by performing comparative transcriptome analysis, quantitative real-time PCR (qPCR) experiments, and phenotypic observations, we examined the possible roles of the *Zur* in the virulence of *P*. *odoriferum*.

## 2. Results

### 2.1. Zur Plays A Negative Role in the Virulence of P. odoriferum BCS7

**Figure 1:**
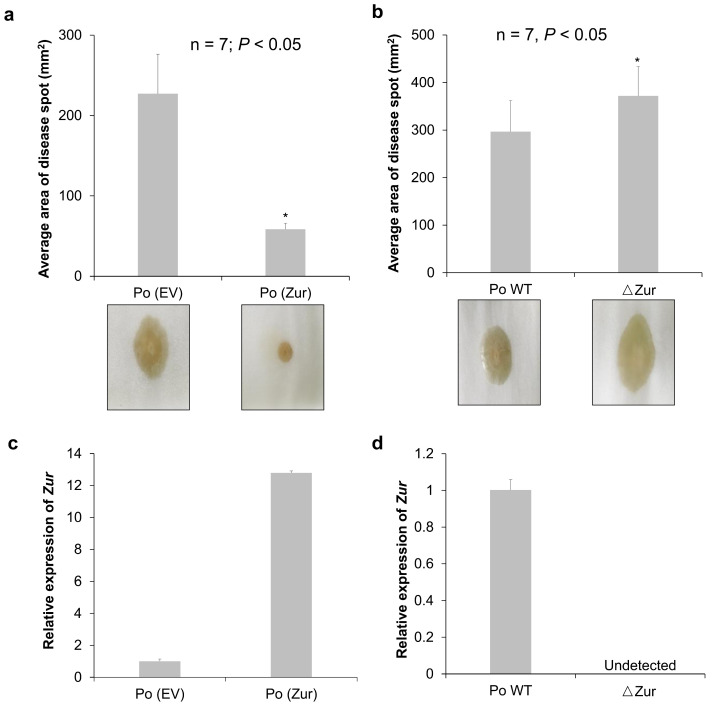
*Zur* contributes to the virulence of *Pectobacterium odoriferum*. (**a**) Virulence of *Zur*-overexpressing strain Po (*Zur*) was measured by examining the average area of the disease spot on Chinese cabbage, with the strain Po (EV) harboring an empty vector as control (n = 7). (**b**) Virulence of the mutant strain of the *Zur* (*ΔZur*) was measured by examining the average area of the disease spot on Chinese cabbage, with the wild-type strain (Po WT) as control (n = 7). (**c**,**d**) These show the relative expression of the *Zur* in the Po (*Zur*) and *ΔZur* compared to their control strains Po (EV) and Po WT, respectively. Each column shows the mean and standard deviation (n = 3). Significant differences with *p* < 0.05 (*t*-test) were labelled with an asterisk. All experiments were repeated three times independently, and similar trends were observed. The results of one experiment are shown.

To identify the role of the *Zur* in the virulence of *P*. *odoriferum* BCS7, *Zur*-overexpression (Po (*Zur*)) and *Zur*-knockout (*ΔZur*) strains were constructed and used to inoculate Chinese cabbage petioles. The lesion area was measured at 24 h post-inoculation for each strain (including the control strains). Plants inoculated with Po (*Zur*) displayed a smaller lesion area compared to those inoculated with the control strain Po (EV) (*p* < 0.05), whereas the *Zur* mutant strain (*ΔZur*) induced a larger lesion area than the wild-type strain Po BCS7 (Po WT) (*p* < 0.05) (Figure 1a,b). *Zur* expression in the Po (*Zur*) and *ΔZur* were confirmed using qPCR (Figure 1c,d). These results suggest that the *Zur* in *P*. *odoriferum* BCS7 plays a negative role in virulence.

### 2.2. Zur does not Affect the Growth of P. odoriferum BCS7

**Figure 2:**
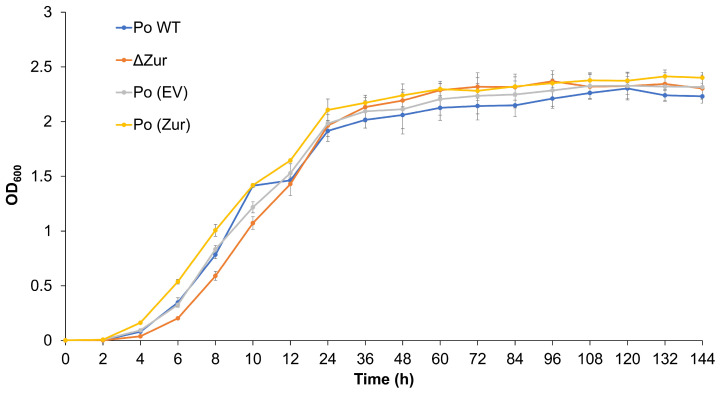
Growth curve over 6 days (144 h) of *ΔZur* and Po (*Zur*). The assay was repeated three times independently, and similar trends were found. The results of one experiment are shown. Values are the mean ± SD (n = 3). Po WT: wild-type strain of Po BCS7, which was used as a control for *ΔZur*; Po (EV): Po BCS7 strain harboring an empty vector, which was used as a control for Po (*Zur*).

To determine whether the effect of the *Zur* on the virulence of *P*. *odoriferum* BCS7 was due to its effects on the bacterial growth, we compared the growth curve of the overexpression and mutant strains, Po (*Zur*) and *ΔZur*, and their corresponding control strains, Po (EV) and Po WT, in standard Luria–Bertani (LB) growth medium. The growth curve was monitored for 144 h until the bacteria entered the stationary phase and the growth of the four strains showed no obvious differences (Figure 2). This indicates that the *Zur* has no effect on the growth of *P*. *odoriferum* BCS7 and that its regulatory role in virulence is probably effected through other mechanisms.

### 2.3. Comparative Transcriptome Analysis of Po (Zur) vs. Po (EV) and ΔZur vs. Po WT

**Figure 3:**
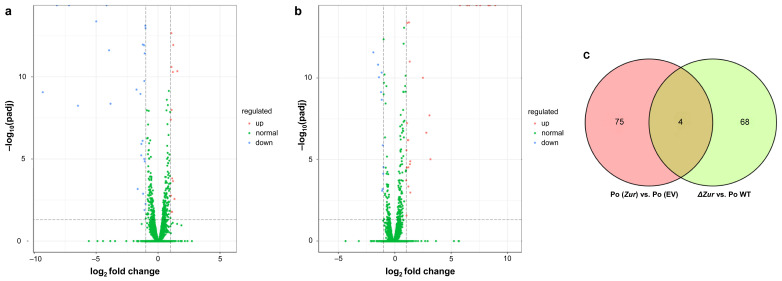
Differentially expressed genes (DEGs) of Po (*Zur*) vs. Po (EV) and *ΔZur* vs. Po WT. Volcano plots of DEGs detected in Po (*Zur*) vs. Po (EV) (**a**) and *ΔZur* vs. Po WT (**b**) are shown. Each dot represents one gene with the *y*-axis showing −log_10_ (padj) and the *x*-axis showing the log_2_ fold change. The red, blue, and green dots represent the upregulated DEGs (padj < 0.05 and log_2_ fold change ≥ 1)), downregulated DEGs (padj < 0.05 and log_2_ fold change ≤−1), and genes that were not significantly regulated, respectively. padj: adjusted *p*-value. (**c**) Venn diagram of DEGs identified in Po (*Zur*) vs. Po (EV) and *ΔZur* vs. Po WT.

To identify the effect of the *Zur* on *P*. *odoriferum* at the transcript level, the transcriptomes of the four strains [Po (*Zur*), Po (EV), *ΔZur*, and Po WT], which were cultured in standard LB growth medium, were sequenced and two comparisons were performed—Po (*Zur*) vs. Po (EV) and *ΔZur* vs. Po WT. Each strain included three biological replicates, and a total of 30.09 Gb raw data and 29.89 Gb clean data were produced. Each sample contained 2.21–2.85 Gb clean data, with a Q20 quality score of 97.67–98.39% and a Q30 of 93.22–94.83% (Table S1). By aligning clean data onto the reference genome of *P*. *odoriferum* BCS7 and conducting a comparative gene expression analysis, genes with an adjusted *p*-value (padj) < 0.05 and fold change (FC) ≥ 2 were identified as differentially expressed genes (DEGs). A total of 79 DEGs (22 upregulated and 57 downregulated) were identified in the comparison between Po (*Zur*) and Po (EV), while for *ΔZur* vs. Po WT, there were 72 DEGs in total, including 56 upregulated and 16 downregulated ones (Figure 3a,b; Tables S2 and S3). The Venn diagrams of the DEGs of these two comparisons showed that they had only four shared DEGs, while most DEGs were distinct between the two groups (Figure 3c). Taken together, these results suggest that both gain-of-function and loss-of-function mutations of the *Zur* modulated the transcriptome of *P*. *odoriferum*, while the effect may be different.

### 2.4. Zur Overexpression Negatively Affects the Flagellum and Cell Motility in P. odoriferum 

**Figure 4:**
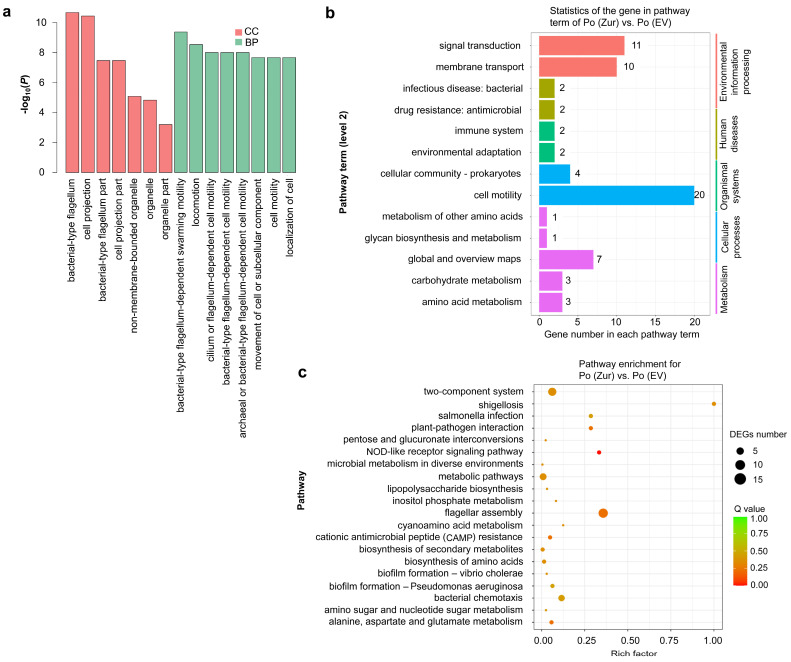
Functional classification of the differentially expressed genes (DEGs) of Po (*Zur*) vs. Po (EV) according to the gene ontology (GO) database and Kyoto Encyclopedia of Genes and Genomes (KEGG) database. (**a**) GO enrichment analysis of the DEGs. All GO terms with a *p*-value threshold of 0.05 in the main categories of cellular component (CC) and biological process (BP) are included, and no DEGs are enriched in the category of molecular function. (**b**) KEGG classification of DEGs. The *x*-axis shows the number of DEGs in each pathway term, and the number of DEGs is indicated at the top of the bar. (**c**) The top 20 pathways enriched by performing KEGG analysis of the DEGs.

To understand which functions of *P*. *odoriferum* were affected by *Zur* overexpression, gene-ontology (GO) and Kyoto Encyclopedia of Genes and Genomes (KEGG) enrichment analyses were performed using the obtained DEGs identified in Po (*Zur*) vs. Po (EV). In the GO enrichment analysis, DEGs in Po (*Zur*) vs. Po (EV) were associated with 15 functional groups belonging to two main categories: cellular components (CC) and biological processes (BP). In the CC category, DEGs were enriched in GO terms such as bacterial-type flagellum, cell projection, and bacterial-type flagellum part, and in the BP category, DEGs were enriched in eight functional groups, all of which were related to motility functions, including bacterial-type flagellum-dependent swarming motility, locomotion, and cilium- or flagellum-dependent cell motility (Figure 4a). In our KEGG pathway annotation results, the pathway term (level 2) of cell motility had the largest number of DEGs (20), followed by signal transduction (11) and membrane transport (10) (Figure 4b). A KEGG enrichment analysis showed that DEGs were largely enriched in the pathways of flagellar assembly, the two-component system, and bacterial chemotaxis (Figure 4c). These analyses showed that *Zur* overexpression regulated the flagellum and cell motility in *P*. *odoriferum*.

**Figure 5:**
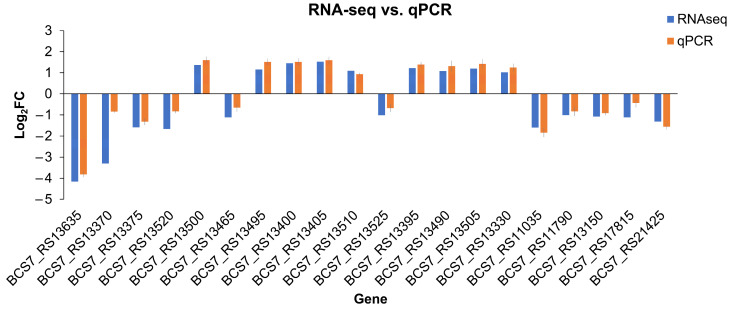
qPCR verification of the expression levels of 20 cell-motility-related DEGs identified in the transcriptomic analysis [Po (*Zur*) vs. Po (EV)]. FC: fold change. Each column of qPCR represents the mean ± SE of three independent biological experiments, and each column of RNA-seq represents the final data of log_2_FC.

To confirm the DEGs identified in our comparative transcriptome analysis, 20 DEGs related to cell motility were selected, and their expression changes were validated using qPCR. The expression patterns of these 20 DEGs were consistent with those of the RNA-Seq analysis (Figure 5), and thus the transcriptome data were confirmed to be reliable. In light of the possible roles of the *Zur* in the flagellum and cell motility of *P*. *odoriferum*, the exact effects were further determined using flagellum staining and a cell motility assay.

**Figure 6:**
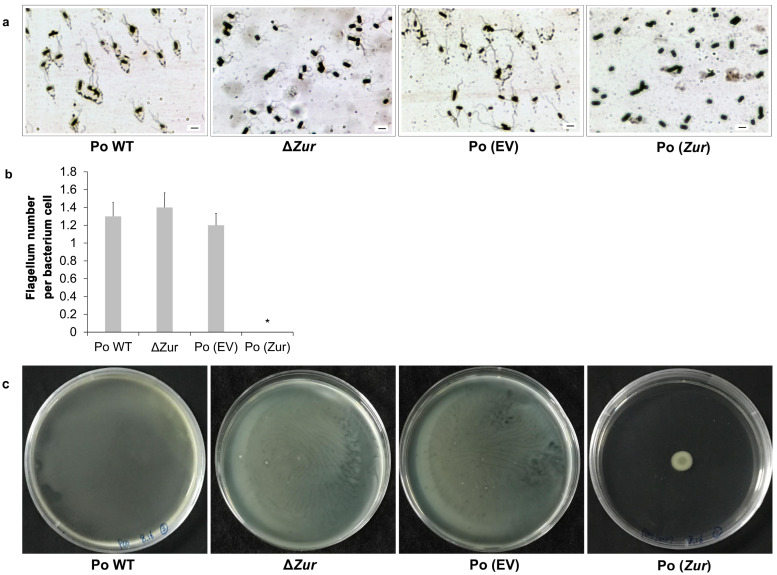
Motility characteristics of *Pectobacterium odoriferum* strains with *Zur* overexpression (Po (*Zur*)) or knockout (*ΔZur*) compared to their respective control strains, Po (EV) and Po WT. (**a**) Flagella stain of each strain. Scale bar = 2 μm. (**b**) Flagellum numbers for each strain were counted and compared. Each column represents the mean ± SE (n = 10), and an asterisk indicates significant difference (* *p* < 0.05, one-way ANOVA). (**c**) The swimming motility of cells was visualized as a halo on a clear background 16 h after patching on LA containing 0.3% agar at 28 °C.

Flagellum staining and quantification of the four strains, Po (*Zur*), Po (EV), *ΔZur*, and Po WT, showed that the Po (*Zur*) exhibited significantly less flagellum (none) in comparison with the control strain Po (EV) (1–2 flagella per bacterium cell) (*p* < 0.05), while the *ΔZur* had no obvious difference in the number of flagella in comparison with the control strain Po WT, both having 1–2 flagella per bacterium cell (*p* > 0.05) (Figure 6a,b). These results indicate that *Zur* overexpression negatively affects the development of flagella in *P*. *odoriferum*, while depletion of the *Zur* does not.

Cell motility assays showed that the Po (*Zur*) had a remarkably reduced halo compared with its control strain, the Po (EV), which nearly swam to the edge of the medium plate (Figure 6c). The strain of the *ΔZur* and its control strain, the Po WT, both swam to the full region of the medium plate and showed no obvious difference in swimming motility. The results suggest that *Zur* overexpression dramatically reduces cell motility in *P*. *odoriferum*, whereas *Zur* knockout does not affect cell motility.

### 2.5. Zur Depletion Affects Metal-Ion Transport Rather Than the Flagellum and Cell Motility in P. odoriferum

**Figure 7:**
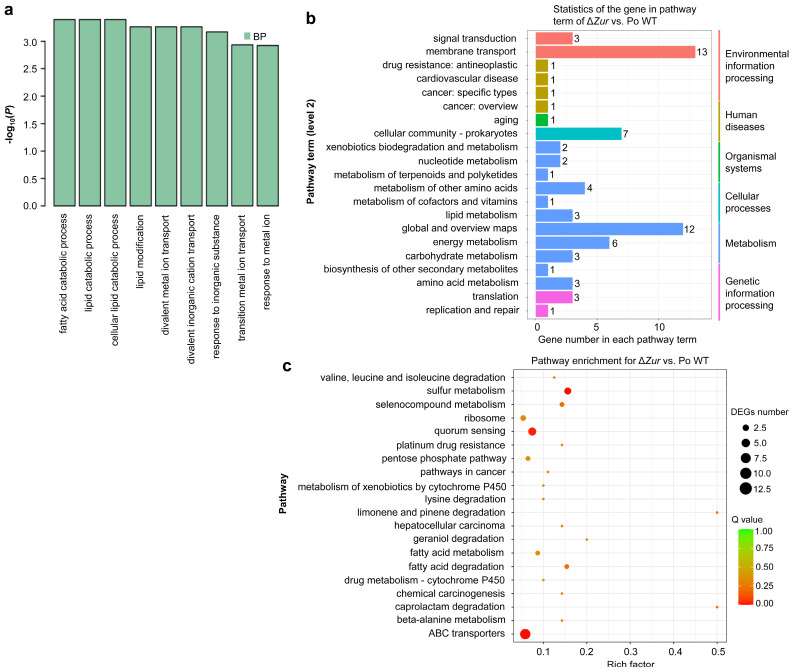
Functional classification of the differentially expressed genes (DEGs) of *ΔZur* vs. Po WT according to the gene ontology (GO) database and Kyoto Encyclopedia of Genes and Genomes (KEGG) database. (**a**) GO enrichment analysis of the DEGs. All the GO terms with a *p*-value threshold of 0.05 in the main category of biological process (BP) are indicated and there is no DEG enriched in the categories of cellular component and molecular function. (**b**) KEGG classification of DEGs. The *x*-axis shows the number of DEGs in each pathway term, and the number of DEGs is indicated at the top of the bar. (**c**) The top 20 pathways enriched by performing KEGG analysis of the DEGs.

*Zur* depletion did not show an effect on the flagellum and cell motility in *P*. *odoriferum* as *Zur* overexpression did (Figure 6). To determine the effect of *Zur* depletion in *P*. *odoriferum* at the transcriptional level, GO and KEGG analyses were also conducted using the DEGs identified in *ΔZur* vs. Po WT. The GO enrichment analysis showed that DEGs were enriched in nine functional terms of the BP category, with divalent-metal-ion transport, response to metal ions, and the fatty acid catabolic process as the major ones (Figure 7a). The KEGG pathway classification showed that, in this comparison, the DEGs were associated with 21 pathway terms (level 2), mainly including membrane transport (13 DEGs), global and overview maps (12), cellular community-prokaryotes (7), and energy metabolism (6) (Figure 7b). In terms of specific pathways, the DEGs were mainly enriched in the pathways of ABC transporters and quorum sensing (Figure 7c). Notably, the DEGs related to metal-ion transport included BCS7_RS09460 (zinc ABC transporter substrate-binding protein ZnuA), BCS7_RS09455 (zinc ABC transporter ATP-binding protein ZnuC) and BCS7_RS09450 (zinc ABC transporter permease subunit ZnuB), which constitute the *znuABC* zinc-uptake system in prokaryotes [5]. These results suggest that, unlike *Zur* overexpression, *Zur* depletion affects pathways such as ion transport rather than the flagellum and cell motility in *P*. *odoriferum*, suggesting that the *Zur* affects the virulence of *P*. *odoriferum* via a dual mechanism.

## 3. Discussion

*Zur* was found in the genome of *P*. *odoriferum*. Research on *Zur* has shown that mutating this gene decreases the virulence of many pathogenic bacteria, including *S*. *enterica*, *X*. *campestris*, *X*. *oryzae* pv. *oryzae,* and *L*. *monocytogenes* [16,20,23,26], except for *S*. *aureus,* whose *Zur* mutant did not show significantly different virulence [15]. To determine the precise function of the *Zur* in the virulence of *P*. *odoriferum*, *Zur* mutant and overexpression strains were constructed. The virulence assay showed that *Zur* overexpression significantly reduced the virulence of *P*. *odoriferum* on Chinese cabbage, while the *Zur* mutant exhibited increased virulence. The regulatory role of the *Zur* on the virulence of *P*. *odoriferum* differs from the *Zur* in previous reports. This indicates that the *Zur* may contribute to virulence through multiple mechanisms.

In *S*. *enterica*, the LD_50_ of the *Zur* mutant increased approximately tenfold when mice were challenged intraperitoneally. The underlying reason for this virulence decrease could be related to the lower transcriptional expression of *fliAZ* in this mutant than in the wild-type strain, as the product of flagellar gene *fliZ* is necessary for the virulence of *Salmonella* serovar Typhimurium cells [16,35]. For *X*. *campestris* [20] and *X*. *oryzae* pv. *oryzae* [23], their *Zur* mutants showed virulence deficiency on host plants, and this could be explained by the decreased production of EPS, which is an important virulence factor in many pathogens and whose production shortage has been related to loss of virulence [23,36]. In our study, *Zur* overexpression in *P*. *odoriferum* reduced the number of flagella and attenuated the cell motility, whereas mutating it did not. The bacterial flagellum is one of the most important organelles. Developed on the bacterial cell surface, it enables bacteria to swim from unfavorable to favorable environments [37,38] and this structure also participates in bacterial pathogen infection through their roles in host cell adhesion, cell invasion, and the secretion of non-flagellar bacterial proteins that are involved in the virulence process [39]. A likely scenario here is that a large amount of the Zur inhibits the virulence of *P*. *odoriferum* by impacting on flagellum production and cell motility, yet the precise mechanism requires further investigation.

In contrast, the loss-of-function mutation of the *Zur* in *P*. *odoriferum* did not cause changes in flagellum production and cell motility, and comparative transcriptome analysis showed that DEGs induced by mutating the *Zur* were not related to flagellum and cell motility. They were mainly involved in metal-ion transport and ABC transporters (Figure 7). Interestingly, DEGs related to metal-ion transport included *BCS7_RS09460* (encoding zinc ABC transporter substrate-binding protein ZnuA), *BCS7_RS09455* (encoding zinc ABC transporter ATP-binding protein ZnuC), and *BCS7_RS09450* (encoding zinc ABC transporter permease subunit ZnuB). These three genes were all upregulated in the *ΔZur*, suggesting a negative regulating role of the *Zur* on the *znuABC* system in *P*. *odoriferum*. This result is unsurprising as the *Zur* has been reported to regulate zinc homeostasis via the *znuABC* zinc-uptake system in bacteria [6]. Zinc is an essential trace element in most organisms, but an excess of it can be toxic. Consequently, to maintain appropriate zinc levels in cells, bacteria have evolved both uptake and export systems that function in concert. Growing evidence has shown that the efficient uptake of divalent metals, including zinc, is critical to successful bacterial infection and virulence [40,41]. Accordingly, the *znuABC* zinc-uptake system, which belongs to the ABC transporter family, is found to be critical for bacterial pathogen virulence, such as full *Salmonella* virulence, in different animal models [42]. As the function of this system is negatively regulated by the Zur protein [9,20,43], it could also be that the increased virulence observed in our *Zur* mutant is due to the upregulation of its zinc-uptake-related *znuABC* genes. This hypothesis requires further investigation.

Taken together, our results indicate that Zur might play a negative role in regulating the virulence of *P*. *odoriferum.* Excessive Zur attenuated virulence-related flagellum and cell motility, while getting rid of the Zur might promote the virulence-related *znuABC* zinc-uptake system. Thus, the underlying mechanisms of the role of the Zur in virulence might be a dose-dependent strategy: a high expression of the Zur attenuates the flagellum and cell motility to reduce virulence, while a low expression of the Zur upregulates the zinc-uptake system of *znuABC* to enhance virulence. As a transcription factor, the Zur regulates the expression of its regulon genes by sensing the intracellular Zn concentrations, but it is important to know what controls the expression of the Zur. Previous studies have provided evidence that the Zur autoregulates its expression in the majority of bacterial species [44,45]. For example, in some species such as *Bacillus anthracis*, the *Zur* autoregulates its expression, being a part of an operon with the *znuB* and *znuC* [6]. It will be of great future interest to perform further experiments to demonstrate the detailed mechanism of the Zur in *P*. *odoriferum* virulence.

## 4. Materials and Methods

### 4.1. Bacterial Strains, Plasmids, and Culture Conditions

All bacterial strains and plasmids used in this study are listed in Table S4. A rifampicin-resistant strain, *P*. *odoriferum* BCS7 (and its derivatives), and *Escherichia coli* were grown on Luria–Bertani broth (LB) or LB agar (LA) plates. The growth temperatures for the *Pectobacterium* and *E*. *coli* strains were 28 °C and 37 °C, respectively. Antibiotics were used at the following concentrations when required: 100 µg/mL rifampin (Rif); 50 µg/mL kanamycin (Km); 50 µg/mL gentamicin (Gm); and 50 µg/mL streptomycin (Sm).

### 4.2. Construction of the P. odoriferum Mutant and the Zur-overexpressing Strain

The *Zur* loss-of-function mutant of *P*. *odoriferum* BCS7 (Po BCST) was constructed by using homologous recombination, as described previously [46], with minor modifications. Briefly, the upstream and downstream regions of the target gene *Zur* were amplified by using PCR with specifically designed primers (Table S5). A kanamycin cassette amplified from pET30a was ligated with these two fragments and cloned into pEX18Gm using an In-Fusion HD Cloning Kit (Takara, China) to yield a recombinant vector. This vector was transferred to Po BCS7 by means of conjugation using *E*. *coli* S17-1λpir. To select strains with gene deletion, transconjugants were plated on LA containing 10% sucrose, Rif, and Km, and the positive colonies were confirmed by means of PCR. To overexpress the *Zur* gene in the Po BCS7 strain, the open reading frame (ORF) of the *Zur,* together with its upstream promoter sequence (500 bp), were amplified using the genomic DNA of the Po BCS7 strain with the specific primers P-Zur-ORF-S1 and Zur-ORF-AS1, and the PCR products were ligated into the linearized plasmid of pBBRIMCS-5 using the In-Fusion HD Cloning Kit (Takara, China). The recombinant construct was transformed into *E*. *coli* DH5α and conjugated to Po BCS7 by triparental mating with *E*. *coli* S17-1λpir. The conjugants were selected on LA containing Rif and Gm and confirmed by means of PCR. The primers used for making the constructions are listed in Table S5.

### 4.3. Pathogenicity Assessment of P. odoriferum with Zur Depletion or Overexpression

The pathogenicity of *Pectobacterium* strains with *Zur* knockout (*ΔZur*) or overexpression [Po (*Zur*)] compared to their respective control strains, Po WT and Po (EV), was determined in vitro by macerating the petioles of Chinese cabbage. For the inoculation, we largely followed the method described by Li et al. [47], with minor modifications. Briefly, 3 μL bacterial suspension (2 × 10^8^ CFU/mL) per site for each strain was used to inoculate Chinese cabbage at 2.5 mm depth, and the rot area for each petiole was measured 24 h later. The virulence of each strain was examined with nine petioles in the pathogenicity assessment (three petioles per Petri dish), and the experiment was repeated three times independently, with similar results.

### 4.4. Transcriptome Sequencing and Analysis

For our transcriptome sequencing, three replicates were included for each strain. Cells of the *ΔZur* and Po (*Zur*) and their respective control strains, i.e., wild-type strain (Po WT) and empty-vector introduced strain [Po (EV)], were cultured in LB with Rif from a 1% dilution of their seed cultures (OD_600_ = 1.0) overnight (16 h). One milliliter of the cultures was centrifuge-pelleted at 8000 rpm for 2 min and washed once with RNA-free water before total RNA extraction using TRIzol. The obtained total RNA was used to prepare sequencing libraries using the NEBNext^®^ Ultra™ Directional RNA Library Prep Kit for Illumina^®^ (NEB, USA) following the manufacturer’s recommendations, and the sequencing was performed on an Illumina NovaSeq 6000 platform. The raw reads of each sample were filtered to produce clean data by means of removing reads containing adapters or poly-N and low-quality reads. We mapped the clean data to the *P*. *odoriferum* BCS7 reference genome (CP009678.1) using STAR software (2.7.10b) [48] and obtained the counts of reads that were uniquely mapped to each gene model. Gene expression levels were normalized as fragments per kilobase of transcripts per million mapped reads (FPKM). DEGs were identified using the DESeq2 package in R [49]. Only genes with an adjusted *p*-value (padj) < 0.05 and an absolute value of log_2_(fold change) ≥ 1 were considered as DEGs. The DEGs were annotated using the GO and KEGG databases. GO enrichment was performed using clusterProfiler software (v3.18.0) with a *p*-value threshold of 0.05 in Fisher’s exact test [50]. The KEGG enrichment was conducted with a hypergeometric test to analyze the enriched KEGG pathway terms of the DEGs, with the whole genome as the background.

### 4.5. Validation of mRNA-Seq by Quantitative Real-time PCR (qPCR)

Twenty DEGs related to cell motility were selected for validation by means of qPCR from the transcriptome data of Po (*Zur*) vs. Po (EV). The cDNA was prepared from the remaining total RNA after transcriptome sequencing by EasyScript One-step gDNA removal and cDNA synthesis supermix (TransGen Biotech, Beijing, China) and subjected to qPCR using TB Green^®^ Premix Ex Taq™ II (Tli RNaseH Plus) (TaKaRa, China) as previously described [51]. RecA was used as the internal reference gene. The fold change in the relative gene expression of targets in samples of Po (*Zur*) compared with Po (EV) was analyzed using the 2^−ΔΔCt^ method [52]. All qPCR primers are listed in Table S5. The experiment was repeated three times independently, with three technical replicates each time.

### 4.6. Phenotypic Assays

Bacterial growth curves: cells of Po (*Zur*), Po (EV), *ΔZur*, and Po WT were cultured overnight in LB medium with Rif for 12–16 h, and the cultures were adjusted to OD_600_ = 1.0 using the original medium. Subsequently, cell suspensions were diluted with 20 mL LB medium containing Rif at a ratio of 1:100 for growth at 200 rpm, and the OD_600_ value was monitored after incubation for 0, 2, 4, 6, 8, 10, 12, 24, 36, 48, 60, 72, 84, 96, 108, 120, 132, and 144 h with an Eppendorf BioPhotometer Plus (Eppendorf, Germany). Flagella staining and observation: bacterial staining was conducted using the silver staining method as previously described [53], and flagella were observed under an optical microscope. Swimming motility assay: a single colony of Po (*Zur*), Po (EV), *ΔZur*, and Po WT was cultured in the LB medium with Rif for 12–16 h to OD_600_ = 1.0. The cell motility of each strain was visualized based on swimming haloes on an LA plate containing 0.3% agar 16 h after 3 μL of the cell suspension was patched onto it and incubated at 28 °C. The experiment was repeated three times with at least three replicates.

### 4.7. Statistical Analysis

A statistical analysis of data differences was performed using an independent *t*-test or one-way ANOVA in IBM SPSS Statistics 20.

## Data Availability

The data supporting the conclusions in this study are available on request from the corresponding author.

## Supplementary Materials

The following supporting information can be downloaded at https://www.mdpi.com/article/10.3390/ijms24129991/s1.
